# Preparation and Characterization of Cellulose Acetate Propionate Films Functionalized with Reactive Ionic Liquids

**DOI:** 10.3390/polym11071217

**Published:** 2019-07-20

**Authors:** Joanna Kujawa, Edyta Rynkowska, Kateryna Fatyeyeva, Katarzyna Knozowska, Andrzej Wolan, Krzysztof Dzieszkowski, Guoqiang Li, Wojciech Kujawski

**Affiliations:** 1 Faculty of Chemistry, Nicolaus Copernicus University in Toruń, Gagarina Street 7, 87-100 Torun, Poland; 2 Normandie Université, UNIROUEN, INSA Rouen, CNRS, PBS, 76000 Rouen, France

**Keywords:** polymer membranes, cellulose acetate propionate, reactive ionic liquid, transesterification reaction, material characterization, water transport

## Abstract

1-(1,3-diethoxy-1,3-dioxopropan-2-ylo)-3-methylimidazolium bromide (RIL1_Br), 1-(2-etoxy-2-oxoethyl)-3-methylimidazolium bromide (RIL2_Br), 1-(2-etoxy-2-oxoethyl)-3-methylimidazolium tetrafluoroborate (RIL3_BF4) ionic liquids were synthesized. Subsequently, the dense cellulose acetate propionate (CAP)-based materials containing from 9 to 28.6 wt % of these reactive ionic liquids were elaborated. Reactive ionic liquids (RILs) were immobilized in CAP as a result of the transesterification reaction. The yield of this reaction was over 90% with respect to the used RIL. The physicochemical properties of resultant films were studied using nuclear magnetic resonance (NMR), scanning electron microscopy (SEM), energy dispersive X-ray (EDX), atomic force microscopy (AFM), and thermogravimetric analysis (TGA). The RIL incorporation influenced the morphology of films by increasing their surface roughness with the rise of RIL content. The thermal stability of CAP-based membranes was dependent on the nature of the ionic liquid. Nevertheless, it was proven that CAP films containing RILs were stable up to 120–150 °C. Transport properties were characterized by water permeation tests. It was found that the type and the amount of the ionic liquid in the CAP matrix substantially influenced the transport properties of the prepared hybrid materials.

## 1. Introduction

Polymer-based membranes have been elaborated for a range of applications, such as biomaterials [1], electrolytes in fuel cells [2], sensors [3], and selective barriers exploited in separation of gas and liquid mixtures (pervaporation, gas separation, reverse osmosis, nano-, ultra-, and micro-filtration) [4,5], as well as for the recovery of metal ions [6]. The design and preparation of a polymeric membrane material with an improved or novel characteristic has been driving the current research in membrane technology [6]. There are various approaches studied, such as the use of polymeric blends, membrane surface modification, membrane cross-linking as well as utilization of the inorganic (e.g., carbon nanotubes—CNTs, metal organic frameworks—MOFs, zeolites) or organic (e.g., ionic liquids—ILs) additives for membrane preparation [7,8,9,10]. Membrane technology is known as energy capable, environmentally approachable, and easy to scale-up, with a large outlook of expanding its utilization with the growing need of employing more sustainable industrial processes. During the design of the membrane material, its selectivity/permeation characteristics have been adjusted and optimized for quite diverse separation tasks. Nevertheless, the membrane fabrication itself could be much more sustainable and greener. To meet that requirement, two aspects of membrane production have to be taken into account: the membrane material and the manufacture process [11]. Concerning the green membrane material, the cellulose is one of the best candidates [12]. However, cellulose can barely be dissolved in common solvents because of strong hydrogen bonds and crystallinity. Chemically modified cellulose in the form of cellulose acetate has been successfully applied in a large scale, for example as planar flat-sheet membranes for forward osmosis and as hollow fibers for seawater desalination by reverse osmosis processes [11].

ILs have drawn special attention from the researchers due to their remarkable properties such as negligible vapor pressures, and high thermal and chemical stability [13]. IL has also been considered as a green solvent [14]. IL is a salt consisting of an organic cation and inorganic/organic anion, possessing a melting point usually below 100 °C [13]. The significant advantage of ILs is the possibility to change their physicochemical properties by altering the cation and anion [15]. Ionic liquids can be classified into two major groups: aprotic and protic ILs [16]. Properties of a given ionic liquid such as hydrophilicity-lipophilicity, viscosity, surface tension, and density depend on the type of cation and anion. Ionic liquids have been examined as a component of a polymeric matrix or polymerization media [17]. The polymer modification with IL was found as a useful method to obtain novel membrane materials with unique composition and material features or good transport and separation properties [18,19]. Membranes containing ILs were successfully utilized for the recovery of alcohols [20], separation of gases [21,22], and as separators in fuel cells [23] or lithium batteries [24].

An increasing interest in the elaboration of the polymeric membranes containing ILs has been widely reported in the literature [2,25,26,27,28,29]. Guo et al. [30] used 1-alkyl-3-methylimidazolium hexafluorophosphate as a plasticizer. Results showed that (C8MIM)(BF4) could successfully replace the traditional molecular plasticizer. The incorporation of ionic liquids also improved permeability [30]. The selection of ILs for the preparation of polymer-IL system in which IL is firmly linked to the polymer matrix is a key challenge, and it depends on the compatibility and the ratio between the IL and polymer [2,31]. IL can be incorporated into the membrane material by mixing the polymer with IL in a suitable solvent prior to the membrane formation [2]. Murakami et al. [32] pointed out that functionalization of cellulose with the polymerizable ionic liquid (PIL) followed by the in situ polymerization results in the interpenetrating polymer-IL network. Another approach to the immobilization of IL is soaking of a native dense or porous polymer matrix with the solution of IL [33]. However, the development of ionic liquids utilization in membrane processes requires solving the problem of ionic liquids leaching [34,35]. Ionic liquids were also incorporated into a membrane matrix in order to increase the membranes stability [36]. Based on the scientific literature, it was found that by addition of ionic liquid, mechanical and thermal stabilities were improved [37,38]. Stability of this type of the membrane depends on the properties of ionic liquid, type of membrane support, and interfacial tension between the membrane support and aqueous phase. Some researchers found that IL can be lost from the membrane. This phenomenon was studied by Zhao et al. [39], and the authors pointed out that two parameters have an impact of the mechanism of the IL loss: membrane compression and ionic liquid extrusion from the membranes possessing large pores. The limit value of pore size that ensures the stability of the material and avoids liquid losses were placed in the range of 100–200 nm [39]. The mentioned mechanism was defined based on the data for membrane prepared with 1-n-butyl-3-methylimidazolium tetrafluoroborate ((bmim)(BF4)) immobilized in the following matrices: polyvinylidene fluoride (PVDF), nylon 6 (N6), and polyethersulfone (PES) microporous membranes. The promising approach to the stable polymer-IL system elaboration is the chemical grafting of IL onto the polymer [40]. Hassan Hassan Abdellatif et al. [40] proposed the sequential synthesis route for IL grafting onto cellulose acetate (CA) in order to minimize the leaching of IL as well as to enhance the membrane performance in the ethyl tert-butyl ether (ETBE) purification using pervaporation. Researchers reported the successful modification of CA with IL containing bromide anion and various cations (imidazolium, pyridinium, and ammonium) confirmed by 1H nuclear magnetic resonance (NMR) analysis. Moreover, the diminished release of IL from the membrane due to the covalent bonds between IL and CA was pointed out. CA-based membranes chemically modified with ammonium-based IL revealed the highest performance in the pervaporative separation of the ethanol-ETBE mixture at 50 °C among all tested membranes. The obtained flux was equal to 0.182 kg m-2 h-1 for the membrane thickness of 5 μm [40]. A preparation of ionic gels is another approach to improve the stability of ionic liquid in the membrane matrix. In this type of materials, the ILs are immobilized in the swollen polymer matrix [41,42]. However, gas separation tests showed that ionic gels were damaged during experiments under the influence of applied pressure [41]. The promising method to minimize the IL leakage challenge is also a formation of membrane based on the chemical modification of polymer with reactive ionic liquid (RIL) proposed in our previous work [43]. The used ester-functionalized RILs consist of cations with ethoxy groups that can react with the acetate or propionate substituents in CAP based on transesterification reaction creating covalent bonds between RILs and polymer. Rynkowska et al. [43] elaborated CAP-based membranes containing 3-(1,3-diethoxy-1,3-dioxopropan-2-yl)-1-methyl-1H-imidazol-ium bromide and used for pervaporative dehydration of 2-propanol. The pervaporation results showed that introduction of RIL into a membrane matrix improves the separation properties of CAP-based membranes. Moreover, the RIL incorporation increases the membrane mechanical properties comparing to native CAP membrane and CAP membranes with commercial plasticizers (TBC—tributyl citrate and ATBC—acetyl tributyl citrate). CAP-RIL membranes showed also higher elongation at break in contrast with CAP-TBC and CAP-TBAC membranes [43].

The main aim of this work is related to the chemical modification of cellulose acetate propionate (CAP)-based membranes using reactive ionic liquids (RILs) containing imidazolium cation, in order to fabricate the polymer material with immobilized IL. The following RILs were used: 1-(1,3-diethoxy-1,3-dioxopropan-2-ylo)-3-methylimidazolium bromide (RIL1_Br), 1-(2-etoxy-2-oxoethyl)-3-methylimidazolium bromide (RIL2_Br), 1-(2-etoxy-2-oxoethyl)-3-methylimidazolium tetrafluoroborate (RIL3_BF4). The imidazolium cation was chosen due to its feasibility and good compatibility with CAP [44,45]. On the other hand, the anions were selected based on the variation in their hydrophobicity/hydrophilicity properties. The novelty of this work was the formation of membrane materials containing ionic liquids firmly linked to the polymer matrix. Furthermore, to have deeper insight into the comprehensive analysis, the physicochemical and material properties were determined using the various spectroscopic and microscopic techniques. The determined features were subsequently referred to the transport performances of elaborated materials.

## 2. Materials and Methods

### 2.1. Reagents

**Figure 1:**
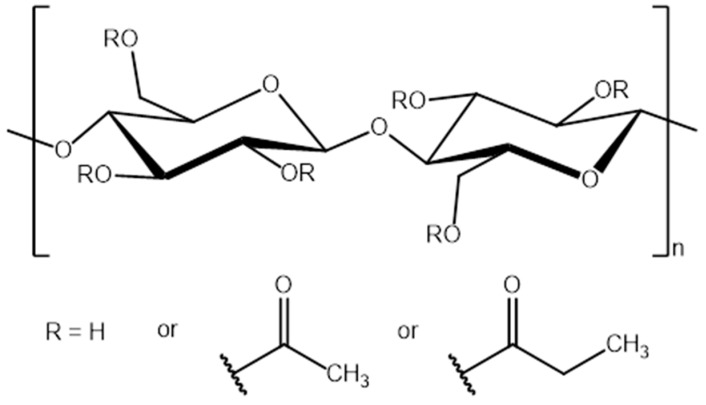
Chemical structure of cellulose acetate propionate (CAP).

The polymer films were prepared using cellulose acetate propionate (CAP-482-20; Eastman, USA; MW = 75,000 g mol-1, where MW is polystyrene-equivalent molecular weights determined using size exclusion chromatography) with acetyl, propionyl, and hydroxyl group content equal to 1.3, 48.0, and 1.7 wt %, respectively (Figure 1). N-methylimidazole (Sigma-Aldrich, Poland), ethyl bromoacetate (Sigma-Aldrich, Poland), sodium tetrafluoroborate (Avantor Performance Materials Poland S.A., Poland), diethyl 2-bromomalonate (abcr GmbH, Karlsruhe, Germany) were used to synthesize RILs. Solvents chloroform, diethyl ether, acetone delivered by Avantor Performance Materials Poland S.A. (Gliwice, Poland) were used as received. Reverse osmosis (RO) deionized water (15 MΩ·cm) was utilized within this study. Methanol and water of Honeywell liquid chromatography-mass spectrometry (LC-MS) grade were used in LC-MS analysis.

### 2.2. Reactive Ionic Liquids (RILs) Synthesis

#### 2.2.1. 1-(1,3-Diethoxy-1,3-Dioxopropan-2-ylo)-3-Methylimidazolium Bromide (RIL1_ Br)

**Figure 2:**
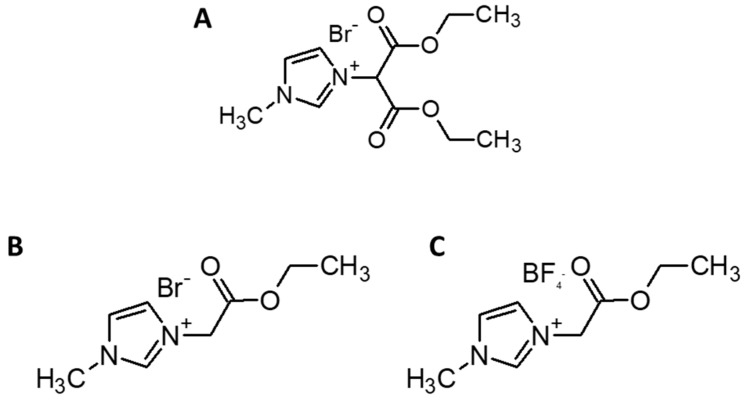
Chemical structure of (**A**) 1-(1,3-diethoxy-1,3-dioxopropan-2-ylo)-3-methylimidazolium bromide (RIL1_Br), (**B**) 1-(2-etoxy-2-oxoethyl)-3-methylimidazolium bromide (RIL2_Br), and (**C**) 1-(2-etoxy-2-oxoethyl)-3-methylimidazolium tetrafluoroborate (RIL3_BF4).

N-methylimidazole (0.80 mL; 10 mmol), chloroform (10 mL), and diethyl 2-bromomalonate (1.70 mL; 10 mmol) were added to a 50 mL round-bottom flask under an argon atmosphere and heated under reflux for 24 h. Subsequently, chloroform was removed using a rotary evaporator, and the remaining yellow liquid was washed three times with 20 mL of diethyl ether. The residues of used solvents were removed under vacuum (66.7 Pa) during 5 h. The yield of RIL1_Br synthesis was equal to 98.8%, and its structure (Figure 2A) was confirmed by NMR analysis [43]. 1H NMR (400 MHz, Bruker Avance III spectrometer, CDCl3) δ ppm 10.55 (s, 1 H), 7.83 (t, J = 1.8 Hz, 1 H), 7.58 (t, J = 1.8 Hz, 1 H), 7.07 (s, 1 H), 4.34 (dqt, J = 15.9, 7.1, 3.7 Hz, 4 H), 4.13 (s, 3 H), 1.33 (t, J = 7.2 Hz, 6 H). 13C NMR (101 MHz, CDCl3) δ ppm 163.11, 138.42, 123.24, 122.99, 64.16, 62.93, 37.12, 13.90.

#### 2.2.2. 1-(2-Etoxy-2-Oxoethyl)-3-Methylimidazolium Bromide (RIL2_Br)

Ethyl bromoacetate (1.67 g; 10 mmol) was added carefully to the 25 mL round-bottom flask containing a solution of n-methylimidazole (0.82 g; 10 mmol) in chloroform (10 mL) and heated under reflux for 24 h. Subsequently, chloroform was removed using a rotary evaporator, under vacuum (0.1 Pa) during 6 h. The yield of RIL2_Br synthesis was equal to 96.4%, and its structure (Figure 2B) was confirmed by NMR analysis. 1H NMR (700 MHz, CDCl3) δ ppm 10.17 (s, 1 H), 7.63 (t, J = 1.8 Hz, 1 H), 7.48 (t, J = 1.8 Hz, 1 H), 5.48 (s, 2 H), 4.07 (s, 3 H), 4.25 (q, J = 7.1 Hz, 2 H), 1.30 (t, J = 7.2 Hz, 3 H). 13C NMR (176 MHz, CDCl3) δ ppm 165.69, 138.08, 123.44, 122.52, 62.60, 49.95, 36.52, 13.67.

#### 2.2.3. 1-(2-Etoxy-2-Oxoethyl)-3-Methylimidazolium Tetrafluoroborate (RIL3_BF4)

A 50 mL flask was charged with 1-(2-etoxy-2-oxoethyl)-3-methylimidazolium bromide (RIL2_Br) (1.25 g; 5 mmol) and acetone (30 mL). Subsequently, sodium tetrafluoroborate (0.55 g; 5 mmol) was added, and the resultant solution was stirred at ambient temperature for 24 h. The white crystalline precipitate of sodium bromide was filtered. Acetone was removed using a rotary evaporator under vacuum (0.1 Pa) during 5 h. The yield of RIL3_BF4 synthesis was equal to 76.5%, and its structure (Figure 2C) was confirmed by NMR analysis. 1H NMR (700 MHz, DMSO-d6) δ ppm 9.08 (d, J = 0.4 Hz, 1 H), 7.73 (dt, J = 6.2, 1.7 Hz, 2 H), 5.24 (s, 2 H), 4.20 (q, J = 7.1 Hz, 2 H), 3.91 (s, 3 H), 1.24 (t, J = 7.1 Hz, 3 H). 13C NMR (176 MHz, DMSO-d6) δ ppm 166.95, 137.79, 122.78, 123.49, 61.98, 49.60, 36.08, 14.07.

### 2.3. Preparation of Cellulose Acetate Propionate-Reactive Ionic Liquids (CAP-RILs) Films

**Figure 3:**
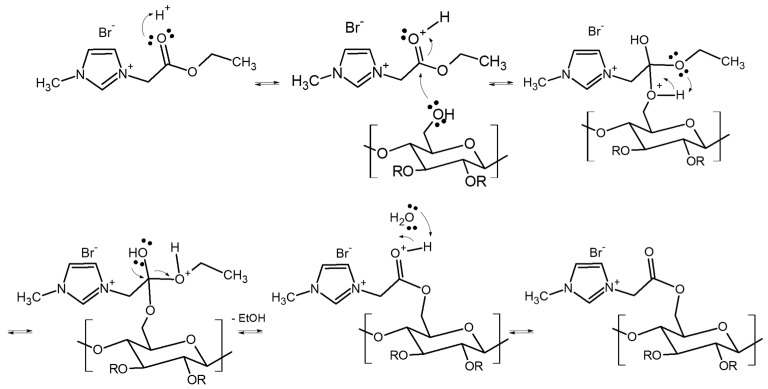
Scheme of the transesterification reaction between CAP and RIL2_Br.

The 10 wt % CAP polymer solution was prepared as follows: 10 g of cellulose acetate propionate powder was dissolved in 90 g of chloroform, and the obtained mixture was stirred at room temperature (21 °C ± 3 °C) during 24 h in order to obtain the homogenous solution. The various amounts of RILs (9; 16.7; 23; and 28.6 wt % of RILs with respect to CAP content) were mixed with the CAP solution in chloroform at the room temperature during 24 h. During this time, the transesterification reaction between CAP and RILs took place (Figure 3), which was subsequently confirmed by the NMR analysis. The transesterification reaction occurred between the propanoyloxymethyl group in CAP and 2-etoxy-2-oxoethyl in RIL. The transesterification reaction starts by the protonation of the carbonyl group by the acid protons, thus activating it towards the nucleophilic attack. Then, the carbonyl group undergoes the nucleophilic attack by the CAP molecule. Subsequently, the proton transfer takes place. The charge displacement from the –OH group to –O+ results in the removal of the ethanol molecule. Finally, the deprotonation of the resultant molecule occurs and the final product of the transesterification reaction is obtained (Figure 3).

The polymer-RIL solution was poured onto a glass Petri dish and placed under the hood at ambient temperature. CAP-RIL dense films were prepared by applying the phase inversion method (i.e., precipitation by solvent evaporation) [46]. CAP-RIL films were left for solvent evaporation up to 48 h in order to obtain dry film samples.

### 2.4. Characterization of CAP-RILs Films

The 1H NMR and 13C NMR spectra of CAP-RIL films were recorded on a 400 MHz Bruker Avance III or 700 MHz Bruker Avance III spectrometer (Bruker, Rheinstetten, Germany) at 25 °C using DMSO-d6 as a solvent. 

The distribution of chemical elements was studied using a scanning electron microscopy (SEM) microscope (LEO 1430 VP, LEO Electron Microscopy Ltd. Cambridge, UK) with an energy dispersive X-ray (EDX) spectrometer (Quantax 200, Bruker AXS Microanalysis GmBH, Germany; with EDX detector XFlash 4010, Bruker AXS Microanalysis GmBH, Germany) at 28 kV. EDX analysis was accomplished for non-sputtered samples. The morphology of the obtained CAP-RIL films was studied by SEM microscope (Quanta 3D FEG) at 30 keV. In that case, prior to SEM analysis, film samples were sputtered with a conductive layer of Au/Pd (80/20 composition) with the thickness equal to 2–6 nm. To obtain a cross-section, the samples for SEM and SEM-EDX analysis were prepared by fracturing the films in liquid nitrogen.

The surface topography of CAP-RIL films was also studied using an atomic force microscope (AFM) with a NanoScope MultiMode SPM System and NanoScope IIIa and Quadrex controller (Veeco, Digital Instrument, UK). The average roughness parameter (Ra) was evaluated based on a tapping mode for the scanned sample area of 10 µm × 10 µm using Nanoscope v6.13 software (Veeco, Digital Instrument, UK). At least three measurements per tested sample were performed in order to obtain reproducibility of the results.

Contact angles for the investigated membranes were measured by implementation of goniometric technique (Attention Theta from Biolin Scientific, Gothenburg, Sweden) using the sessile drop method. Measurements were performed for water as a liquid after 5 s of equilibration at room temperature.

The thermal stability of CAP-RIL films was tested using a thermogravimetric analyzer TGA Q 500 (TA Instruments, USA) from 25 °C to 800 °C under the nitrogen atmosphere (heating rate 10 °C/min and nitrogen flow rate 90 mL/min).

### 2.5. Conversion Degree of RIL

The exact yield of the reaction between CAP and RIL was determined by applying the following procedure using an LC-MS system. This device was chosen due to the greater sensitivity in relation to NMR spectroscopy. Weighed pieces of studied membranes were sonicated with 2000 μL of water for 20 min. 20 μL of the obtained solutions were diluted with 1500 μL of methanol and examined using LC-MS system, measuring the area of the 241 m/z peak in positive selected ion monitoring (SIM) mode. Shimadzu Liquid Chromatograph Nexera-i LC-2040C 3D Plus coupled with Shimadzu Mass Spectrometer LCMS-2020 was used in this experiment. Moreover, Phenomenex Kinetex 2.6 μm C18 100A 50 × 4.60 mm chromatographic column was used in order to reduce a potential interference impact and a noise value.

### 2.6. Water Permeation Tests

**Figure 4:**
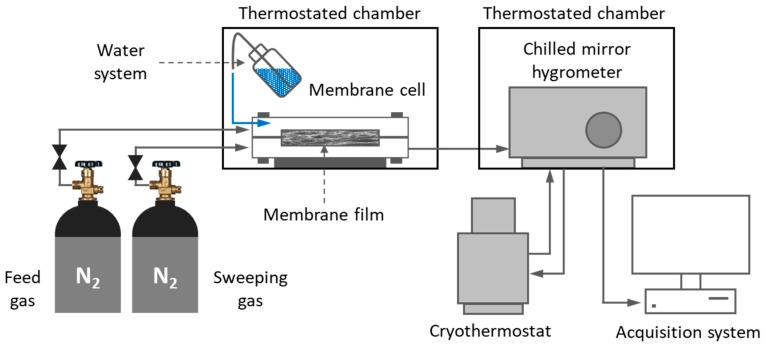
Scheme of water permeation setup.

Water permeation measurements were performed at 25 ± 1 °C implementing a laboratory-made experimental rig (Figure 4). Prior to the tests, the membrane sample (active area of the membrane 2.5 cm^2^) was sealed in the permeation cell and dried by circulating nitrogen (Alphagaz 2, Air Liquide^®^, Paris, France) on both sides of the film until the lowest constant value of dew point (referred to the lowest amount of moisture in the system) was reached (ca. −70 °C). Then, liquid water (used as a feed) was injected into the upstream compartment while the downstream compartment was continuously swept out by the stream of dry nitrogen. Because of the differences in water activities on both sides of the membrane, water molecules diffused across the membrane. The amount of vapor transported into the downstream compartment was monitored by using a chilled mirror hydrometer (General Eastern Instrument, Elcowa^®^, Mulhouse, France) connected to the data acquisition system. The experiments were done at water vapor activities of feed stream increasing up to the saturation vapor pressure. The data were collected until the system reached the stationary state.

## 3. Results

### 3.1. Nuclear Magnetic Resonance (NMR) Characterization

NMR enables us to investigate and detect the interactions between the imidazolium ring and its local vicinity based on the changes of the chemical shifts in the NMR spectra [47]. The magnitude of chemical shifts is affected by the substitution of a given atom or functional group in the given molecule by another one, and it strongly depends on the substituents nature and position in respect to the discussed nucleus [42]. Chen et al. [47] pointed out that protons of the imidazolium ring are notably sensitive to its environmental changes, which can be observed in NMR spectra [47]. Pure RILs and resultant CAP-RILs films were characterized using 1H NMR analysis in order to confirm the effectiveness of the transesterification reaction between the polymer and studied ionic liquids.

**Figure 5:**
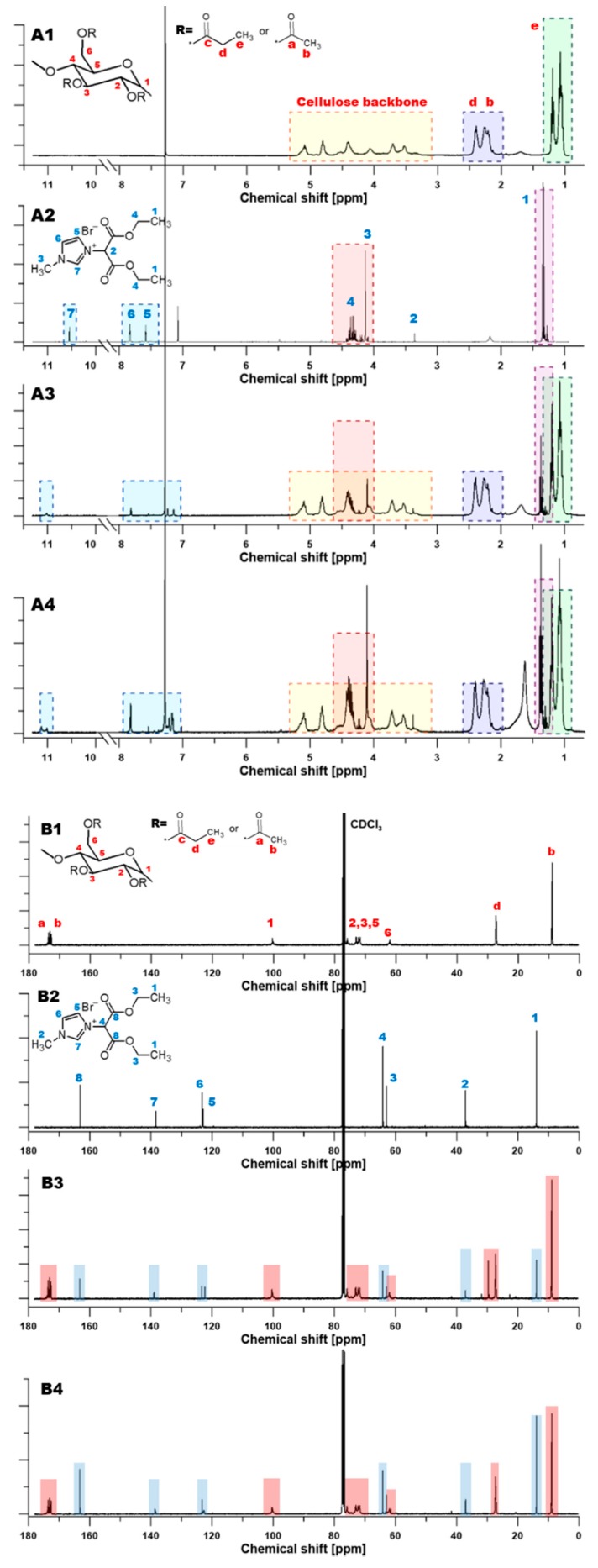
^1^H (**A**) and ^13^C (**B**) NMR spectra of pristine (**A1**,**B1**) CAP and RIL1_Br (**A2**,**B2**), modified CAP with 9 wt % of RIL1_Br (**A3**,**B3**) and modified CAP with 23 wt % of RIL1_Br (**A4**,**B4**).

High resolution proton NMR is an excellent analytic tool to monitor various reactions. The proton signal peaks for CAP were as follows: δ 3.2–5.9 ppm (protons of the cellulose backbone, 7H), δ 2.3 ppm (methyl protons of acetyl, 3H), δ 1.1 ppm (methyl protons of propionyl, 3H), and δ 2.2 ppm (methylene protons of propionyl, 2H) (Figure 5A).

13C NMR spectrum of pure CAP presents the shifts δ 170–173 ppm assigned to carbonyl carbons, whereas the shifts δ 0–30 ppm correspond to the signals of the acyl carbon (Figure 5B). δ 60–110 ppm correspond to the signals of the anomeric glucose unit (AGU) carbonate region. The peak at 63.0 ppm is related to the C6 carbon in the acetyl group. The peaks at 78.0 ppm and 101.2 ppm and are assigned to C4 and C1 in the glucose unit. The observed cluster of resonance peaks of C2, C3, and C5 carbons is explained by their heavy overlap between 71–74 ppm and associated with high DS sample [48,49].

The presence of RILs influenced the 1H NMR spectra of CAP-RIL films (Figure 5). The peaks of CAP below 5.5 ppm did not change after the transesterification reaction. It can be seen that the H2 proton at C2 carbon of the imidazole ring is de-shielded, therefore, the H2 proton signal is shifted for the CAP-RIL films with respect to pure RIL (Table 1), e.g., the chemical shift of the H2 proton in CAP-9-RIL1_Br from 10.55 to 11.02 ppm in comparison to RIL is observed (Table 1) [50]. Simultaneously, one of the protons at C4 and C5 carbons in the imidazole ring is shielded and shifted from 7.58 to 7.24 ppm (Figure 5A, Table 1). Shin et al. [51] also noted that only one of the H4, H5 protons shifts as a result of the change from ethyl to butyl substituent [51]. The aforementioned changes prove the successful transesterification reaction between the RIL and CAP.

The quantitative assessment of the transesterification reaction yield between the propanoyloxymethyl group in CAP and 2-etoxy-2-oxoethyl in RILs was also performed based on the substitution degree (DS) and discussed from the point of view of RIL content (Table 2, Equation (1)). The occurrence of a substitution reaction possesses a significant impact on the characteristics of cellulose derivatives, like crystallinity, solubility, thermal, and mechanical properties [52,53,54].

DS for the CAP-RIL films was estimated taking into account the ratio of the proton integrals of the imidazolium ring and AGU according to Equation (1) [37]:

(1) $$\mathrm{DS}=\frac{S1}{S2}$$

where, S1 is the integral of the resonance (7.81 ppm) of the imidazolium ring proton, and S2 is the integral of the resonance (5.10 ppm) of the proton of AGU [52].

The maximum theoretical degree of substitution (DS_max_) was calculated as the ratio of RIL and anhydroglucose (AGU) molar masses taking into account the weight of RIL and CAP used to prepare CAP-RIL films according to the following equation (Equation (2)):

(2) $${\mathrm{DS}}_{\max}=\frac{M_{\mathrm{RIL}}m_{\mathrm{RIL}}}{M_{\mathrm{AGU}}m_{\mathrm{CAP}}}$$

where, MAGU is the average molar mass of the AGU unit in CAP (325.67 g mol^−1^), MRIL is the molar mass of the ionic liquid, and mCAP and mRIL is the weight (g) of CAP and RIL respectively, in a given CAP-RIL film. MAGU was calculated using the data provided by the producer (Eastman, USA), concerning the acetyl, propionyl, and free OH groups content in CAP equal to 1.3 wt %, 48.0 wt %, and 1.7 wt %, respectively. Generally, the primary carbon atom at C6 position (Figure 5A) is the most reactive CAP substituent. In the case of RIL1_Br possessing two ester groups, it is assumed that the transesterification reaction takes place only with one ester group due to the steric hindrances. Therefore, the degree of substitution (DS) and DSmax was calculated taking into account the 1:1 molar ratio between CAP and RILs.

**Figure 6:**
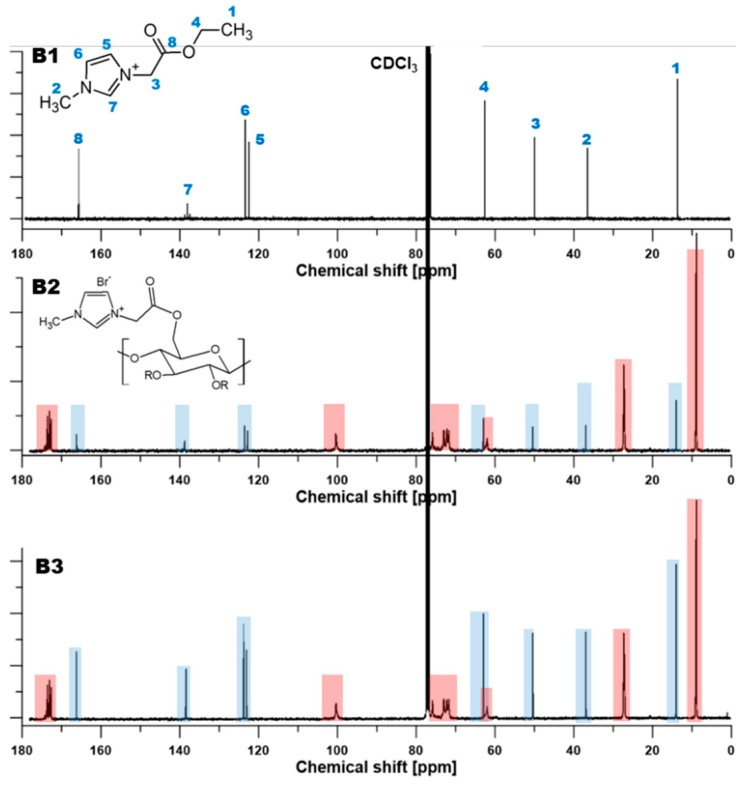
^1^H (**A**) and ^13^C (**B**) NMR spectra of pristine RIL2_Br (**A1**,**B1**), modified CAP with 9 wt % of RIL2_Br (**A2**,**B2**) and modified CAP with 23 wt % of RIL2_Br (**A3**,**B3**).

**Figure 7:**
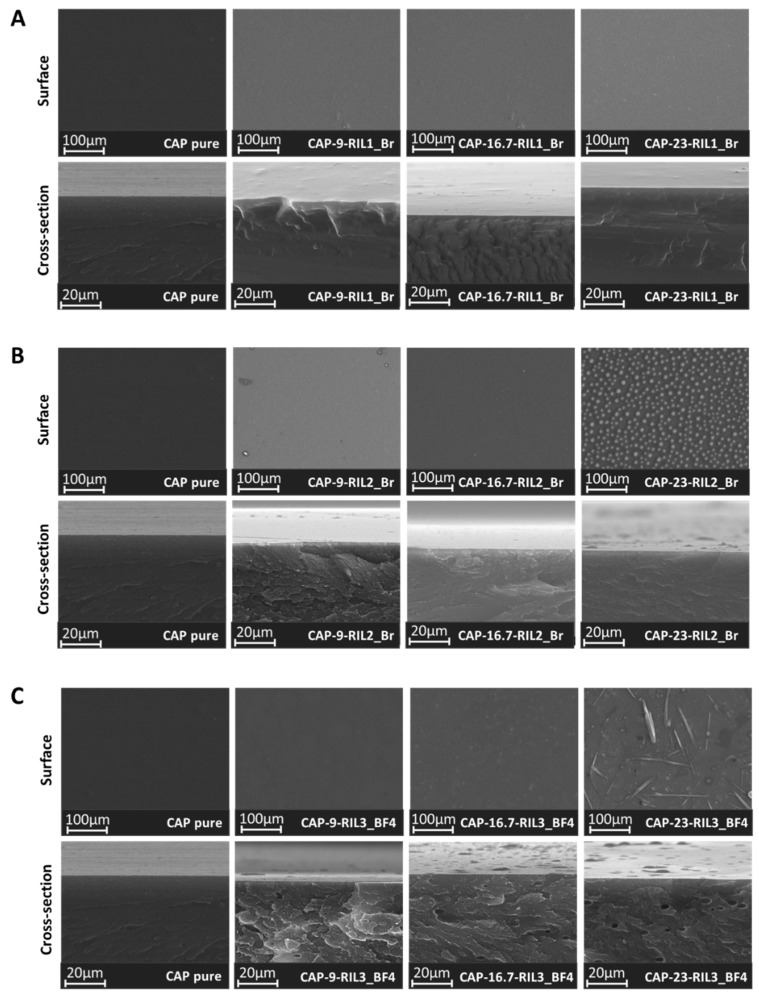
SEM images of surface and cross-section of (**A**) CAP-RIL1_Br, (**B**) CAP-RIL2_Br, and (**C**) CAP_RIL3_BF4 films.

It can be noticed that the reaction between CAP and RIL led to the various partial substitution of ester groups in CAP. The calculated degree of substitution was equal to 0.087 and 0.283 for CAP-9-RIL1_Br and CAP-23-RIL1_Br, as well as 0.027 and 0.076 for CAP-9-RIL2_Br and CAP-23-RIL2_Br, respectively (Table 2). The comparison of the obtained DS with the DSmax reflects the amount of RIL that was attached to the polymer chain. For example, the DS = 0.087 for CAP-9-RIL1_Br indicated that 88% of the ionic liquid was chemically substituted to the CAP. The calculated DS values for CAP-RIL1_Br films are comparable to the values of the maximum theoretical degree of substitution that testifies to the high-efficiency of the transesterification reaction between IL and CAP [43]. It was assumed that the unreacted RIL can form the RIL-reach domains observed by the SEM and AFM analysis (Figure 7 and Figure S1, respectively). The low value of DS presented in Table 2 can be explained by the hindrance steric effect as well as by the reactivity of the anion. Heinze et al. [55] as well as Pinkert and co-workers [12] pointed out that an aldehyde moiety can react with ILs at the reactive proton in position 2 by the generation a covalent bond between the IL and the aldehyde function from the cellulose unit [12]. This reaction is predominantly noticeable during the interaction with ionic liquid containing an imidazolium ring (e.g., 1-ethyl-3-methylimidazolium acetate [55]) and possessing an acetate group as a counter ion. On the other hand, when a cation is less reactive, e.g., chlorine or bromide, C-1 peaks (100.3 ppm) of the reducing end groups can still be seen [55] (Figure 5; Figure 6). This is related to the fact that chloride or bromide containing ionic liquids do not bind so efficiently to the reducing end of the carbohydrate [12,55]. The results obtained in this work are in accordance with the abovementioned findings from the scientific literature [12,55]. Although the chemical modification between polymeric matrix and ionic liquids took place, the effectiveness was not so high. To summarize, the selected ionic liquids were anchored within the CAP membrane significantly influencing the material, physicochemical, and transport properties of the formed materials.

### 3.2. Subsection Conversion Degree of RIL

The degree of RIL conversion after the transesterification reaction with CAP was additionally examined using the LS-MS system. The yield of the reaction between CAP and RIL was determined as ratios of the difference between calculated peak areas per membrane masses for unreacted and studied membranes versus areas per masses for unreacted membranes. Yields amounted to 99% and 91% for CAP-9-RIL1_Br and CAP-23-RIL1_Br membranes, respectively.

### 3.3. Morphology of CAP-RILs Films

SEM images of pristine CAP and CAP RIL1_Br are presented in Figure 7. Results of SEM analysis of pristine CAP and CAP-based films modified with RILs proved the formation of dense films. The morphological study of CAP-RIL films revealed the decreased homogeneity of the film structure with increasing content of RIL. In general, at the low RIL content, the film surface roughness is comparable to that of the native CAP, while an increase of RIL content results in the change of the film surface, which can evidence the differences in the substitution ability of CAP with RIL. This was reflected by the presence of RIL-rich and CAP-rich domains on the film surface as well as by the appearance of unconnected small pores, particularly in the case of CAP-RIL2_Br and CAP-RIL3_BF4 (Figure 7) films as a result of the increased ionic liquid content up to 23 wt %. In the case of RIL1_Br, the increase of ionic liquid content up to 28 wt % results in the appearance of the IL domains on the membrane surface. It should be also remarked that values of DS (Table 2) for CAP-9-RIL2_Br and CAP-23-RIL2_Br are equal to 35% and 33% respectively, thus RIL2_Br is not entirely bound to CAP. Therefore, the presence of unbound RIL (RIL-rich domains) can be expected (Figure 7).

Taking into account the fact that all CAP-based membranes were prepared following the same procedure, the IL migration to the film surface and thus, the appearance of the IL agglomerates on the membrane surface, can be enhanced by the differences in the polymer–RIL interactions and RIL viscosity. Such tendency was noticed by de los Rios for Nylon^®^ and Mitex-based supported liquid membranes with 1-butyl-3-methylimidazolium hexafluorophosphate (BMIM)(PF6), 1-butyl-3-methylimidazolium tetrafluoroborate (BMIM)(BF4), and 1-butyl-3-methylimidazolium bis{(trifluoromethyl)sulfonylimide, (BMIM)(Tf2N) [56]. The researchers observed the ILs agglomerates with various size on Mitex and Nylon membrane surfaces as a result of differences in ILs viscosity and fluidity. The procedure of membranes elaboration was identical for all polymer-IL systems [56]. It was pointed out that the amount of IL agglomerated on the membranes surface increases with the rise of the IL viscosity according to the following sequence: (BMIM)(PF6) > (BMIM)(BF4) > (BMIM)(Tf2N) [56]. The researchers also stated that the homogeneity and the good compatibility between Nylon and studied ILs was correlated with their hydrophilic nature in contrast to the hydrophobic Mitex membranes doped with the studied ionic liquids. The interactions between hydrophilic ILs and Mitex were limited due to the highly hydrophobic nature of Mitex polymer [56].

Xi et al. [57] indicated that the addition of the high loading of the 1-ethyl-3-methylimidazolium hexafluorophosphate (EMIM)(PF6) to waterborne polyurethane (WPU) membranes results in the microphase separation. At high (EMIM)(PF6) content (over 10 wt %) the IL-rich domains were formed on the membrane surface which was attributed to the incompatibility between the IL and WPU matrix [57].

The results of the SEM analysis were also evaluated taking into consideration the polymer/ionic liquid amount ratio used to elaborate a given film. In principle, the transesterification reaction could theoretically take place between three ester groups in the AGU unit and one ester group in RIL2_Br and RIL3_BF4 ionic liquids. Therefore, the ratio between CAP and RIL required for the total substitution of CAP ester by RIL2_Br or RIL3_BF4 is 1 to 3 that would correspond even to the 28.9 wt % of the ionic liquid in the polymer matrix. The content of ionic liquid in CAP films exceeding this ratio may result in the release of ionic liquid excess and the formation of RIL-rich domains on the membrane surface. In fact, RIL-rich agglomerates can be seen for CAP-RIL2 and CAP-RIL3 films containing at least 23 wt % of RIL (Figure 7B,C, respectively). These results suggest that the substitution reaction was not completed and only two ester groups in AGU could possibly be substituted by RIL2_Br or RIL3_BF4. Thus, the ratio between the polymer and the ionic liquid is 1:2, which corresponds to around 21 wt % of the ionic liquid in the polymer matrix. These considerations are in accordance with the results of the SEM analysis.

Assuming that the transesterification reaction can take place between two ester group in RIL1_Br and three ester groups in AGU unit, the polymer: RIL1_Br ratio is 2:3, which corresponds to 21 wt % of RIL1_Br in the polymer matrix. CAP film with the RIL1_Br content equal to 23 wt % does not have visible changes on the film surface that can confirm the full substitution and the high compatibility between CAP and RIL1_Br, which is in agreement with a high degree of substitution (Table 1). On the other hand, in the case of RIL1_Br, the ratio of polymer to RIL1_Br could be 1:3, taking into account the steric hindrance that might result from the branched structure of the ionic liquid. The further increase of RIL1_Br content up to 28 wt % led to the change of the membrane surface and appearance of heterogeneity on its surface (Figure 7).

**Figure 8:**
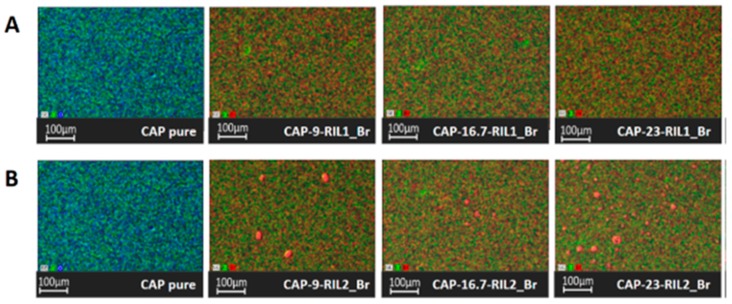
SEM- energy dispersive X-ray (EDX) images of film surface: (**A**) CAP and CAP-RIL1_Br and (**B**) CAP and CAP-RIL2_Br films (green—carbon atoms, red—bromine atoms, and blue—oxygen atoms).

Figure 8 presents the SEM-EDX mapping of native CAP and modified CAP-based films. In the EDX images of pristine CAP film, the characteristic elements assigned as carbon and oxygen can be observed. The presence of carbon and oxygen results from the chemical composition of the polymer. The new signal of bromine was detected by EDX analysis for CAP-RIL1_Br and CAP-RIL2_Br. In the case of CAP-RIL2_Br films containing 23 wt % and 28.6 wt % of ionic liquid, the RIL-rich domain on the film surface was also observed (Figure 8B). In the case of CAP-RIL_BF4, the boron atom cannot be detected due to the limitation of SEM-EDX analysis, therefore, the mapping for CAP-RIL_BF4 films was not shown. SEM-EDX is more suitable for heavy atoms analysis, therefore the light atoms such as boron are not detected, or they give false signals. The comparable results related to the monitoring of ILs behavior after modification with the polymeric matrix were presented by de los Rios et al. [56]. The authors observed that the studied imidazolium-based ionic liquids are homogeneously distributed within Nylon^®^ membranes which was reflected by the SEM-EDX analysis recorded at the membrane cross-section [56]. Xi et al. [57] reported the homogenous dispersion of 1-ethyl-3-methylimidazolium hexafluorophosphate (EMIM)(PF6) in the waterborne polyurethane (WPU) membranes up to the 10 wt % of (EMIM)(PF6). The increase of the (EMIM)(PF6) content above 10 wt % resulted in the formation of IL-rich domains that were dispersed in the WPU matrix [57].

The film surface morphology was also studied and visualized by using AFM analysis and discussed based on the roughness average parameter (Ra) (Figure S1). The performed AFM analysis revealed that the addition of RIL influences the surface roughness of tested films (Figure S1). The increase of the surface roughness of CAP-RIL films with the rise of the RIL content was observed. Such an effect was the most pronounced in the case of RIL3_BF4 incorporation to the film matrix. Therefore, only these results are shown in Figure S1. Three-dimensional (3D) images of CAP-RIL3_BF4 films reflect the increase of the surface roughness. This result is related to the appearance of the RIL-rich domains on the CAP-based film surface, which is in accordance with the results obtained by SEM (Figure 7) analysis. In the case of the sample containing the highest level of IL, i.e., CAP-23-RIL3_BF4, the hills in the x direction were observed. It was related to the lower viscosity of the RIL3_BF4 IL in the comparison to polymeric matrix and higher interaction between IL and AFM probe. Xi et al. [57] applied AFM analysis and observed an increase of the surface roughness of the waterborne polyurethane (WPU) membranes with increasing IL content. It was found that the presence of (EMIM)(PF6) disrupts the polymer chains order resulting in the rougher WPU-(EMIM)(PF6) membranes compared to the flat surface of pristine WPU. For example, the value of Ra parameter increased from 0.81 nm for pure WPU up to 6.89 nm for WPU membrane with 15 wt % of (EMIM)(PF6) [57]. Moreover, the authors highlighted that ILs appeared on the surface of the polymeric membranes and generated thin films due to the low viscosity of the used ILs. The researchers also found that the incorporation of high IL content leads to the phase separation and thus, the occurrence of the IL-rich domains of (EMIM)(PF6) precipitated on the membrane surface [57].

### 3.4. Thermogravimetric (TGA) Analysis

**Figure 9:**
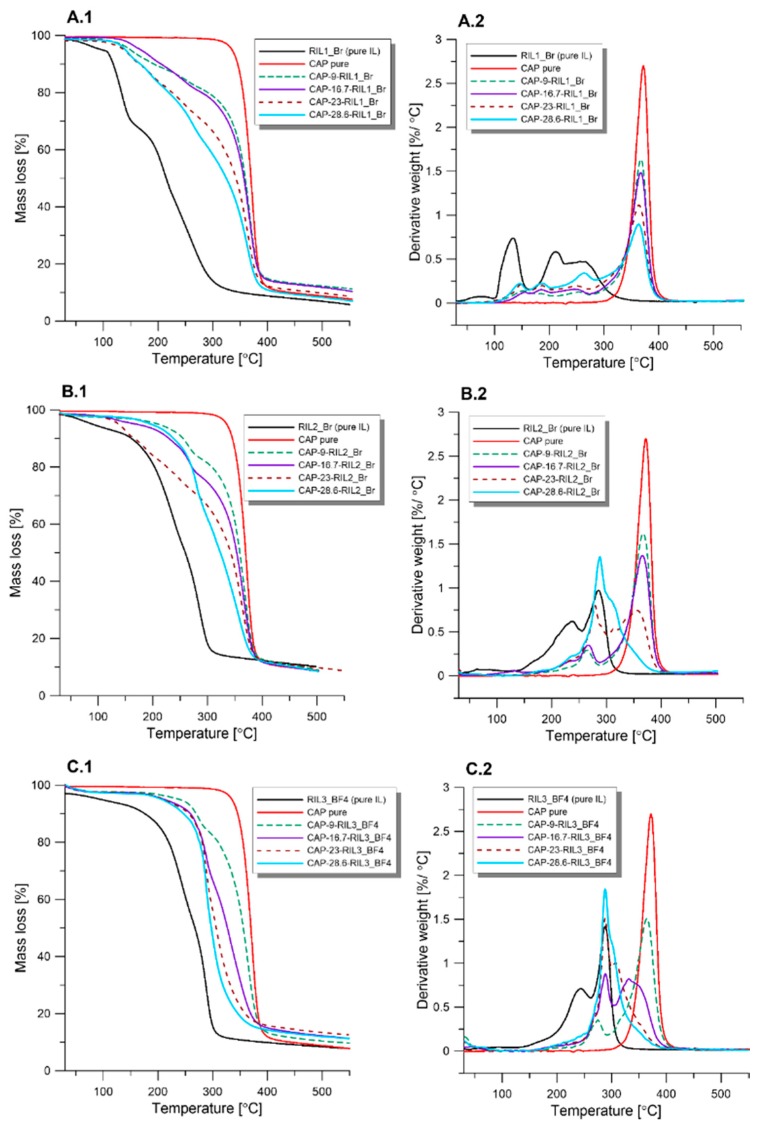
Thermogravimetric analysis of CAP-RIL films. **A1**, **B1**, **C1**-TGA and **A2**, **B2**, **C2**-DTG curves.

Figure 9 presents thermogravimetric (TGA) and derivative thermogravimetric (DTG) curves for the pristine CAP, pure RILs, and CAP-RIL films. The degradation of pure CAP films takes place in one stage, and it corresponds to the simultaneous loss of acetyl and propionyl groups from the cellulose backbone and the pyrolysis of cellulose structure which occurs in the temperature range 300–420 °C. In the case of pure RIL1_Br, RIL2_Br, and RIL3_BF4 numerous decomposition stages can be observed (Figure 9A2–C2).

**Figure 10:**
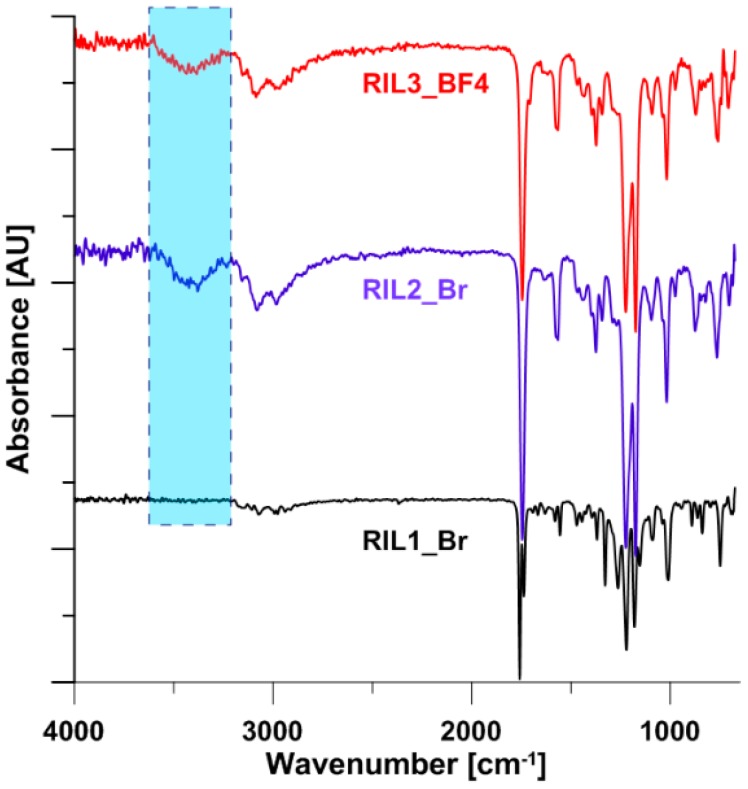
Infrared spectra for pure ionic liquids.

TGA analysis of pure ionic liquids revealed the presence of moisture, indicated by the peak at around 100 °C. These observations were supported and proved by the infrared spectra (Figure 9). The presence of moisture was referred to by the peaks observed at ca. 3390 cm^−1^. The highest level of moisture has been detected for RIL1_Br and slightly smaller for RIL2_Br. On the other hand, no such observation was found for RIL3_BF4 ILs both on the TGA-DTG (Figure 9C1,C2) and infrared spectra (Figure 10). The absorbed moisture by the RIL1_Br and RIL2_BF4 was related to their high hygroscopic properties. The degradation stage between 190–280 °C corresponds to the decomposition of imidazolium side chains [58,59]. Erdmenger et al. suggested that the degradation of imidazolium-based ionic liquid containing tetrafluoroborate anion leads to the creation of alkylimidazole, alkyl fluoride and BF3 [60]. RIL2_Br possesses similar thermogram to RIL3_BF4, whereas RIL1_Br decomposition starts at lower temperature values compared to RIL2_Br and RIL3_BF4. In Figure 10A1–C1 it can be seen that RIL3_BF4 possesses a higher temperature of degradation compared to RIL1_Br and RIL2_Br. This difference is mainly related to the less branched structure of RIL1_Br and RIL2_Br imidazolium cation (Figure 2).

Raj et al. [61] suggested that the nature of the anions influences the thermal stability of an ionic liquid. Cao et al. [62] observed that strongly coordinating anions, such as halogen (Cl–, Br– or I–), decrease the IL degradation temperature, whereas anions which are weakly coordinated (e.g., BF4–, PF6–) will lead to a higher IL decomposition temperature. The obtained results for CAP-RIL films revealed the drop of film thermal stability in comparison to pure CAP due to the incorporation of ionic liquid, which is in accordance with the results found by Rynkowska et al. [19] for CAP-based firms containing 1-methyl-3-(4-vinylbenzyl)-1H-imidazole-3-ium chloride polymerizable ionic liquid. In the Figure 9A2–C2 it can be also noticed that the peak intensity at around 370 °C diminished with increasing content of RIL in the film matrix and the peak position shifts to lower temperature value. Moreover, the increase in peak intensity at around 290 °C was found due to the increasing content of RIL in the film matrix. The highest increase of this degradation peak simultaneously with the reduction of the degradation peak at 370 °C was observed in the case of CAP films with the 28.6 wt % of RIL2_Br and RIL3_BF4 (Figure 9B2,C2, respectively). Such behavior is related to the presence of the unbounded RIL, which was revealed by SEM and SEM-EDX analysis (Figure 7; Figure 8, respectively).

It was found that the maximum degradation temperature (T_max_) at the first degradation peak of CAP-RIL1_Br membranes is around 150 °C. Whereas in the case of CAP-RIL2_Br and CAP-RIL3_BF4 it is equal to 200 °C (Figure 9). Hence, the membranes thermal stability is found to be satisfactory for their potential further application in the pervaporation or gas separation processes, carried out at a temperature usually up to 110 °C.

### 3.5. Membrane Surface Hydrophilicity

**Figure 11:**
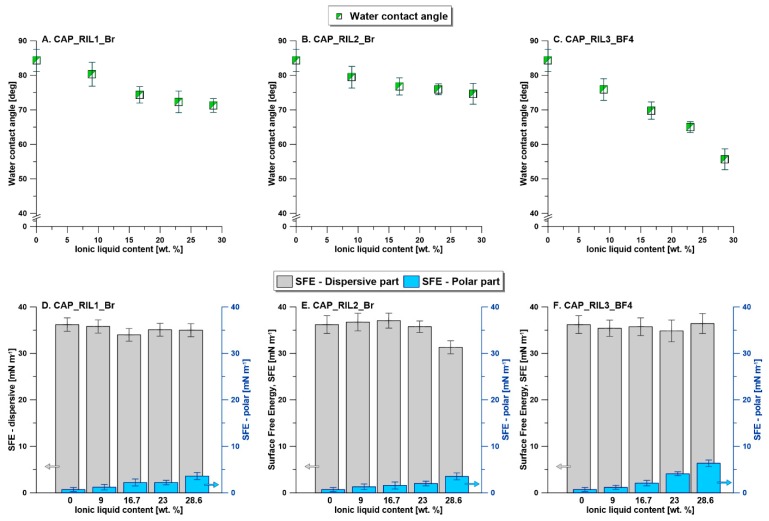
Physiochemical characterization of the formed polymeric materials modified with ILs. **A**–**C**—water contact angle, **D**–**F**—surface free energy.

In the formation of a new type of materials, an important part of research is related to their physiochemical properties. In the presented work, water contact angle (WCA) and surface free energy (SFE) with partial components (e.g., polar and dispersive parts) were determined (Figure 11). The surface free energy was calculated based on the Owens, Wendt, Rabel, and Kaelble (OWRK) method [63]. In the case of all functionalized membranes with ILs, the presence and amount of ionic liquid has a significant impact on the material properties. Due to the differences in hydrophilicity of the ionic liquids, the substantial influence on contact angle values was observed (Figure 11A–C). In all cases, with the increasing amount of ILs in the polymeric matrix, a reduction of WCA has been found. For the pristine CAP material, the contact angle value was equal to 84.3 ± 3.1°. However, the strongest effect was found in the case of CAP-based membranes containing RIL3_BF4 ionic liquid, WCA _CAP_28.6_RIL3_BF4_ = 55.7 ± 2.1° (Figure 11C).

For the membranes modified with RIL1_Br and RIL2_Br the contact angle values for the samples containing 28.6 wt % of RIL were equal to 71.3° ± 2.0° and 74.6° ± 3.0°, respectively (Figure 11A,B). The obtained data revealed the efficiency of the polymeric membranes modification from the point of view of material properties. Taking into account surface free energy, an important impact was noticed for the polar part of the overall SFE (Figure 11D–F). This observation was related to the fact that hydrophilicity influences polar interaction the most. An addition of hydrophilic ILs to the polymeric matrix influenced the dispersive part of SFE to a much less extent (Figure 11).

### 3.6. Mechanical Properties

The influence of RILs incorporation into the polymer matrix on the mechanical properties of CAP-based films was also investigated by tensile tests and was evaluated in terms of Young’s modulus (E), stress at break (σ_max_), and elongation at break (ε_max_) parameters.

**Figure 12:**
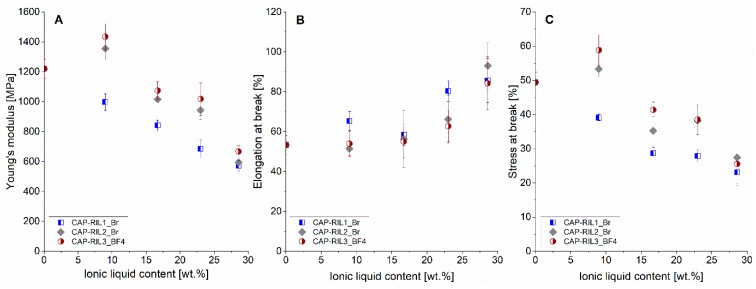
Young’s modulus (**A**), stress at break (**B**), and elongation at break (**C**) of the CAP-based membranes modified with RILs.

The pristine CAP film is brittle, which is reflected by Young’s modulus, stress at break, and elongation at break values equal to E = 1,220 ± 63 MPa, σ_max_ = 49 ± 3 MPa, and ε_max_ = 53% ± 5%, respectively (Figure 12). The comparison of the results for CAP-based membranes with the pure CAP revealed the significant change of the mechanical properties as a result of the RILs incorporation. It was observed that the increasing RIL content enhances the elasticity and simultaneously reduces the brittleness of CAP-based materials (Figure 12), which is reflected by the increasing elongation at break value and decreasing values of stress at break and elastic modulus. The elastic modulus value decreased from 1220 MPa for pure CAP to around 600 MPa for CAP-RIL membranes containing 28.6 wt % of RIL. Simultaneously, the elongation at break value increased from 53% for pure CAP to around 85% for CAP-RIL films containing 28.6 wt % of RIL. The decrease of stress at break values as a function of RIL content can be seen from 49 to around 25 MPa for pure CAP and CAP-28.6-RIL films, respectively. Such behavior corresponds to the reduced rigidity of CAP-based films with simultaneous improvement of their elongation. The obtained results of the studied mechanical properties of the CAP-RIL films testifies the plasticization of CAP membrane materials by RIL.

The plasticization phenomena of polymer membranes by ionic liquid were also observed by Schmidt et al. [64]. The authors found that the incorporation of imidazolium-based ionic liquids led to the plasticization of Nafion^®^ 117 matrix. This fact was confirmed by the diminished Young’s modulus value compared to the pristine Nafion^®^ 117 membrane [64], and it was explained by the presence of the butyl and hexyl alkyl side chains in the used ionic liquid. It was also pointed out that the incorporation of ionic liquids with side chains characterized by high flexibility weakens the hydrogen bonds between sulfonic groups in the Nafion membrane [64]. The similar phenomenon of polymer membrane plasticization by ionic liquid incorporation was reported by Chen et al. [65] for sulfonated poly(ether ether ketone) (SPEEK) membranes doped with 1-ethyl-3-methylimidazole tetrafluoroborate or 1-butyl-3-methylimidazole methanesulfonate ionic liquids.

### 3.7. Transport Properties

Taking into account the application of the prepared materials, it is essential to characterize not only their mechanical stability but also their transport features. The transport properties of the pure CAP and CAP containing RILs were studied by permeation tests with liquid water. Taking into account kinetics of water permeation, the experimental curves of water flux were fitted considering the possible water plasticization effect in CAP-based membranes in the function of local water concentration C based on Equation (3) [66].

(3) $$D=D_{0}e^{\gamma C}$$

where, D_0_ is the diffusion coefficient when water concentration tends to have the value equal to 0, γ is the plasticization factor, and C is the local water concentration in the material [66].

**Figure 13:**
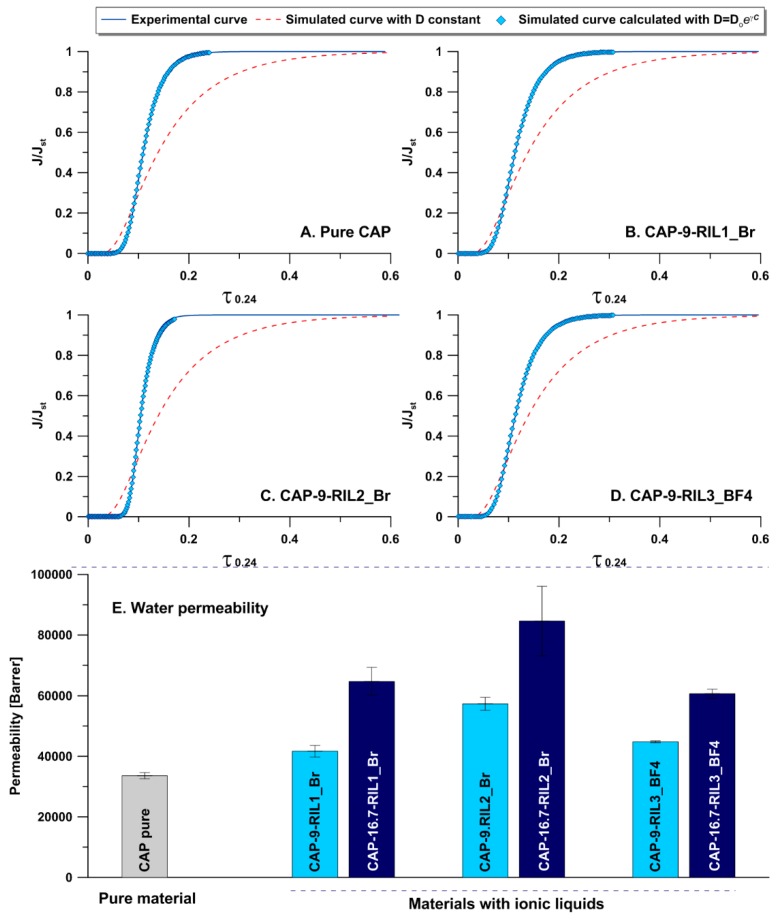
Transport properties of pristine and modified materials, **A**–**D** curves of experimental and simulated water flux for investigated materials (**A**-CAP, **B**-CAP-9-RIL1_Br, **C**-CAP-9-RIL2_Br, **D**-CAP-9-RIL3_BF4) and water permeability as the function of ILs content (**E**). Barrer is equal to 3.35 x 10^−16^ mol m m^−2^ s^−1^ Pa^−1^ in SI unit.

In the Figure 13A–D, the curves for water flux in a dimensionless scale of flux and time are presented for pristine polymeric material (Figure 13A) and modified membranes containing 9 wt % of all types of the investigated ionic liquids (Figure 13B–D). In all cases, very good fitting of the experimental data to the simulated values has been found for water fluxes of CAP-RILs materials. The determined values of permeability for water were gathered and presented in Figure 13E. It was noticed that the presence and, more importantly, the amount of ionic liquid, influenced the transport properties. The reduction of water barrier properties was observed with the increasing value of RIL. On the other hand, the type of ionic liquid has no important impact on the permeability. The most permeable material was the CAP modified with RIL2_Br (Figure 13E). This phenomenon can be related to the fact that IL possessing one reactive group was modified with a much higher effectiveness due to the lack of hindrance steric effects. The lowest transport behavior was observed for the polymeric samples with RIL3_BF4 addition. In that case, the variations are associated with the presence of a counter ion with different chemistry and hydrophobicity. The more profound influence was observed for the samples containing higher levels of ionic liquid, i.e., 16.7 wt %. Based on the literature, the polymeric films, e.g., polystyrene (which adsorb only little amount of water), behave at low vapor pressures in the accordance with Fick’s law, and permeation velocity is inversely proportional to film thickness. For higher pressures where increased sorption of water takes place, deviations from Fick’s law were observed [67,68].

## 4. Conclusions

In the presented work, the efficient method based on the transesterification reaction for the chemical modification of CAP-based films with various ester-functionalized imidazolium-based reactive ionic liquids (RILs) has been developed. Ionic liquids were synthesized and their structures were identified by NMR spectra. It was found that the molecular structure of RILs possesses the predominant effect on the prepared CAP-based films. The conversion degree of RIL in the transesterification reaction was in the range 88%–96% for RIL1_Br ionic liquid, however it was much lower for RIL2_Br (ca. 35%). This low value of conversion degree results mainly from the hindrance steric effects. Unreacted ionic liquid formed RIL-reach domains observed clearly using AFM and SEM analyses. The ionic liquids with a less branched imidazolium-based cation as well as with the weaker coordination and less hydrophilic anion provided the superior thermal stability of CAP-based film materials. Water permeation transport properties of the fabricated materials were determined and referred to RILs structure. It was found that type of the ionic liquids (type of anion) as well as the amount of the ILs in the polymeric matrix has a direct impact on the transport properties.

## Figures and Tables

**Table 1:** The signals position of H2, H4, and H5 protons of the imidazole ring based on NMR spectra of CAP-RIL1_Br and CAP-RIL2_Br films.

Films	Signals Position of a Proton (ppm)	
	H2	H4 and H5
Pure RIL1_Br	10.55	7.58
CAP-9-RIL1_Br	11.02	7.24
CAP-23-RIL1_Br	11.01	7.21
Pure RIL2_Br	10.17	7.48
CAP-9-RIL2_Br	10.56	7.21
CAP-23-RIL2_Br	10.58	7.24

**Table 2:** The degree of substitution (DS) calculated according to 1H NMR spectra for pure RILs and CAP-RIL films.

	Integral of Resonance (-)	
Films	CAP-9-RIL1_Br	CAP-23-RIL1_Br
S1	1.00 (at 7.81 ppm)	1.00 (at 7.82 ppm)
S2	11.43 (at 5.10 ppm)	3.53 (at 5.09 ppm)
DS	0.087	0.283
DS_max_	0.099	0.295
Films	CAP-9-RIL2_Br	CAP-23-RIL2_Br
S1	1.00 (at 7.41 ppm)	1.00 (at 7.24 ppm)
S2	36.88 (at 5.07 ppm)	13.10 (at 5.07 ppm)
DS	0.027	0.076
DS_max_	0.076	0.229

## Supplementary Materials

The following are available online at https://www.mdpi.com/2073-4360/11/7/1217/s1, Figure S1. AFM analysis (3D profile) of pristine CAP pure and CAP-RIL3_BF4 films.
