# Supplementary material for: Preparation and Characterization of Cellulose Acetate Propionate Films Functionalized with Reactive Ionic Liquids
Source: Polymers (Basel). 2019 Jul 20;11(7):1217. doi: 10.3390/polym11071217 (PMC6680812; doi:10.3390/polym11071217)
Supplement: Supplementary file 1 [file polymers-11-01217-s001.pdf]

Supporting Information

Preparation and characterization of cellulose acetate propionate films functionalized with reactive ionic liquids

Joanna Kujawa[1], Edyta Rynkowska [1,2], Kateryna Fatyeyeva [2], Katarzyna Knozowska [1], Andrzej Wolan [1], Krzysztof Dzieszkowski [1], Guoqiang Li [1], and Wojciech Kujawski [1,*]

1 Nicolaus Copernicus University in Toruń, Faculty of Chemistry, 7, Gagarina Street, 87-100 Toruń, Poland.
2 Normandie Univ, UNIROUEN, INSA Rouen, CNRS, PBS, 76000 Rouen, France
* Correspondence: corresponding author: (W. Kujawski), phone: +48 56 611 43 15, fax: +48 56 611 45 26; email: wojciech.kujawski@umk.pl

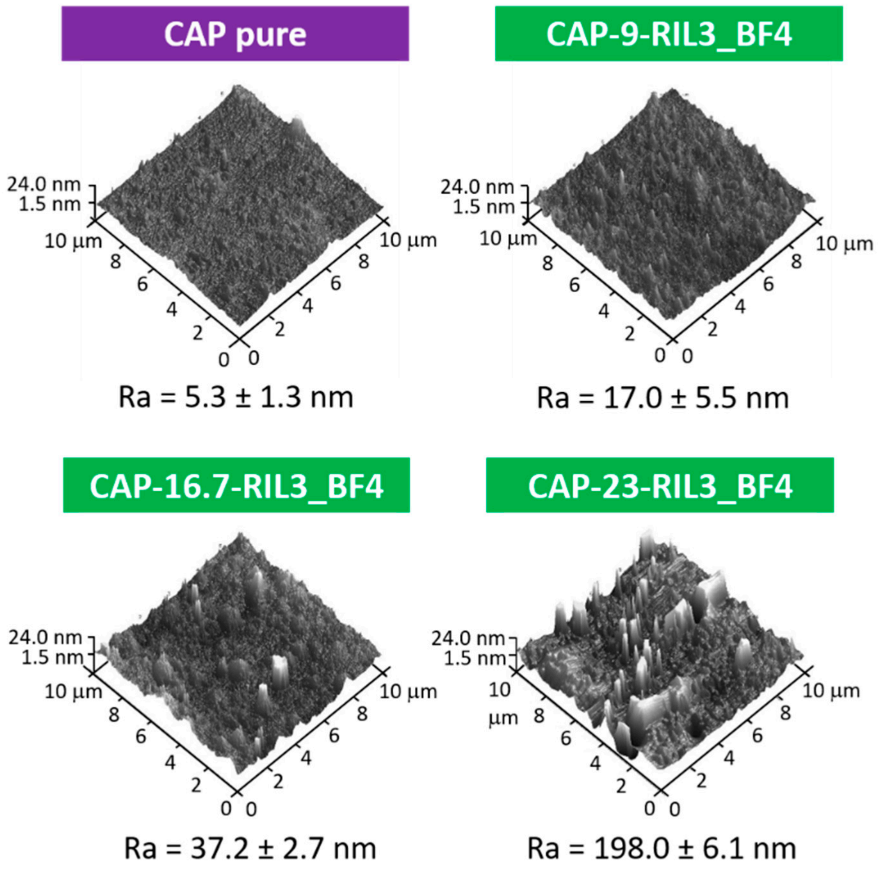

Figure S1. AFM analysis (3D profile) of pristine CAP pure and CAP-RIL3_BF4 films.
